# Psychosocial markers of age at onset in bipolar disorder: a machine learning approach

**DOI:** 10.1192/bjo.2022.536

**Published:** 2022-07-18

**Authors:** Sorcha Bolton, Dan W. Joyce, Katherine Gordon-Smith, Lisa Jones, Ian Jones, John Geddes, Kate E. A. Saunders

**Affiliations:** Department of Psychiatry, University of Oxford, Warneford Hospital, UK; Department of Psychiatry, University of Oxford, Warneford Hospital, UK; and Oxford Health NHS Foundation Trust, Warneford Hospital, UK; Department of Psychological Medicine, University of Worcester, UK; National Centre for Mental Health, Cardiff University, UK

**Keywords:** Bipolar affective disorders, childhood experience, psychosocial interventions, statistical methodology, aetiology

## Abstract

**Background:**

Bipolar disorder is a chronic and severe mental health disorder. Early stratification of individuals into subgroups based on age at onset (AAO) has the potential to inform diagnosis and early intervention. Yet, the psychosocial predictors associated with AAO are unknown.

**Aims:**

We aim to identify psychosocial factors associated with bipolar disorder AAO.

**Method:**

Using data from the Bipolar Disorder Research Network UK, we employed least absolute shrinkage and selection operator regression to identify psychosocial factors associated with bipolar disorder AAO. Twenty-eight factors were entered into our model, with AAO as our outcome measure.

**Results:**

We included 1022 participants with bipolar disorder (μ = 23.0, s.d. ± 9.86) in our model. Six variables predicted an earlier AAO: childhood abuse (β = −0.2855), regular cannabis use in the year before onset (β = −0.2765), death of a close family friend or relative in the 6 months before onset (β = −0.2435), family history of suicide (β = −0.1385), schizotypal personality traits (β = −0.1055) and irritable temperament (β = −0.0685). Five predicted a later AAO: the average number of alcohol units consumed per week in the year before onset (β = 0.1385); birth of a child in the 6 months before onset (β = 0.2755); death of parent, partner, child or sibling in the 6 months before onset (β = 0.3125); seeking work without success for 1 month or more in the 6 months before onset (β = 0.3505) and a major financial crisis in the 6 months before onset (β = 0.4575).

**Conclusions:**

The identified predictor variables have the potential to help stratify high-risk individuals into likely AAO groups, to inform treatment provision and early intervention.

Bipolar disorder is a multi-component mental health disorder characterised by recurring episodes of depression and mania, with a population prevalence of 1–4%.^1^ The clinical trajectory of bipolar disorder is highly variable, with phenomenological and biological heterogeneity contributing to differences in illness course and prognosis.^2^ This makes accurate and timely diagnosis challenging, with patients reporting an average diagnostic delay of almost a decade.^3^ This delay is associated with poorer prognosis, including greater symptom severity and increased suicidality.^4,5^

Recent research has aimed to reduce clinical heterogeneity by demarcating more homogenous subgroups of patients with bipolar disorder, with the aim of improving diagnostic accuracy and refining appropriate treatment options.^6^ It has been proposed that age at onset (AAO) may be a key variable in delineating these subgroups.^7^ Meta-analytic results indicate a differing clinical trajectory between early- and late-onset bipolar disorder, with an early AAO associated with longer delays to treatment, greater severity of depressive episodes, increased suicide risk, increased rates of hospital admission and higher levels of comorbid anxiety and substance misuse.^8,9^ Additionally, initial evidence suggests that there is genetic homogeneity within AAO subgroups and heterogeneity between groups.^10–12^ Despite this, no known research has comprehensively investigated potential psychosocial predictors of AAO.^13,14^ Identifying the risk factors that likely interact with various susceptibility genes to influence bipolar disorder AAO has the potential to inform diagnosis and targeted approaches for early intervention.

## Objectives

We employed a supervised machine-learning approach to build a model examining which psychosocial factors are individually and collectively associated with bipolar disorder AAO. Potential predictors were selected based on their availability in our data-set and possible relevance to bipolar disorder AAO based on prior research. As the data was retrospective, we selected variables that could be reasonably considered as present ‘pre-onset’. These included: family history of suicide, psychiatric and/or affective disorders;^15–17^ alcohol use;^18,19^ drug use;^20,21^ poor premorbid social and work adjustment;^14,22,23^ low educational attainment;^23^ personality traits and temperament;^24–26^ childhood trauma or abuse;^8,27–30^ and stressful life events.^31,32^

## Method

Our study used data from the UK Bipolar Disorder Research Network cohort (BDRN; www.bdrn.org), which is an on-going programme of research into the genetic and non-genetic determinants of bipolar disorder and related mood disorders. All procedures contributing to this work comply with the ethical standards of the relevant national and institutional committees on human experimentation and with the Helsinki Declaration of 1975, as revised in 2008. All procedures involving human patients were approved by a Heath Research Authority NHS Research Ethics Committee (approval number MREC/97/7/01) and all participating NHS Trusts and Health Boards. Written informed consent was obtained from all participants. The data used in the current analysis was accrued from February 2002 to June 2015, and analysed in 2021.

Participants were recruited throughout the UK via NHS services and advertisements through patient support organisations. Inclusion criteria were aged 18 years or over, able to provide written informed consent, met DSM-IV criteria for bipolar disorder, and onset of mood symptoms was before the age of 65 years. Individuals were excluded if they experienced affective illness only because of substance use or medical illness, or were biologically related to another study participant.

### Measures

#### Diagnosis

Best-estimate main lifetime diagnosis was made according to DSM-IV criteria based on in-depth interview with the Schedules for Clinical Assessment in Neuropsychiatry,^33^ and review of psychiatric and primary care case notes, where available.

#### Outcome measure

The primary outcome variable was AAO of bipolar disorder, defined as the age at first clinically significant impairment owing to manic or depressive symptoms. Signs of clinically significant impairment included arguments and/or fights, missed work and/or job loss, treatment referral, the use of lithium or neuroleptics for treatment of manic symptoms, disrupted work or social life, police involvement, family breakdown and psychotic features.

#### Candidate predictors

Twenty-eight predictors (see Supplementary Appendix 1, Section 1.1 available at https://doi.org/10.1192/bjo.2022.536 for full details) were considered. These were selected based on availability in our data-set and potential relevance to bipolar disorder AAO based on prior research. These were: family history of affective disorders, psychiatric disorders and/or suicide; any known sexual and/or physical and/or emotional childhood abuse occurring before the age of 16 years; average number of units of alcohol consumed per week in the year before bipolar disorder onset; regular use of cannabinoids or unspecified non-prescription drugs in the year before bipolar disorder onset; highest level of educational attainment; poor premorbid work and social adjustment (evaluated with the Modified Operational Criteral Symptom Checklist Details and History Questionnaire^34,35^); trait neuroticism (assessed with the neuroticism subscale of the Eysenck Personality Questionnaire Revised^36^); schizotypal personality traits (self-report Kings Schizotypy Questionnaire^37^); cyclothymic, depressive, irritable, hyperthymic and anxious temperament (evaluated with the Temperament Evaluation of Memphis, Pisa, Paris and San Diego Auto-Questionnaire^38^) and significant life events (11 ‘yes/no’ questions from the Brief Life Events Questionnaire^39,40^).

#### Demographics

Age at interview and individuals’ highest level of occupation were recorded. These were not considered as potential predictor variables as they did not specifically relate to pre-bipolar disorder onset. For individuals’ highest occupation, responses were grouped into ‘professional’, ‘non-professional’, ‘never worked’, ‘student’ and ‘unknown’.

### Statistical analysis

The R code used for data pre-processing and analysis is openly available via the Open Science Framework.

#### Data pre-processing

Analysis was conducted in R version 4.0.3 (2020-10-10)^41^ for Mac OS (R Foundation for Statistical Computing, Vienna, Austria; https://www.r-project.org/), using the ‘glmnet’ package version 4.1.1 for the main analysis,^42,43^ along with multiple helper packages (listed in Supplementary Appendix 1, Section 2.1 with references); figures were produced with the ‘ggplot2’ package version 3.3.5.^44^ Missing variables were removed with the listwise-deletion method, with analysis conducted on this data-set. The data-sets with versus without missing data removed were not statistically significantly different from one another (Supplementary Appendix 1, Section 2.2). Pre-processing steps for the full sample included (a) log transforming our outcome variable, AAO, so that age was correctly modelled as a positive number in analyses^45^ (Supplementary Appendix 1, Section 2.3); (b) filtering out data collected pre-2008 as not all questionnaires were administered before this date; (c) dummy coding all categorical variables (with *K*-1 levels per variable) and (d) scaling numeric dependent variables with *z*-score standardisation. We examined the correlations between all 28 predictor variables. Pearson's correlation coefficients ranged from small (±0.21) to moderate (0.68) effect sizes according to Cohen's rule of thumb (Supplementary Appendix 1, Section 2.4). The variables that were most highly correlated were those relating to dimensions of personality and temperament, which are known to be traits that cluster. Accordingly, as these traits cannot be considered in isolation, we retained them in the model building process to preserve ecological validity.

Sociodemographic characteristics of the sample were described with mean and s.d. for continuous variables, and absolute and relative frequencies for categorical variables. We randomised the full sample into a model development (70%, *n* = 717 of total sample) and a held-out validation set (30% of sample, *n* = 305). This 70:30 split allowed us to have a sufficiently large training set for the purposes of model development, while maintaining an adequately large sample size for out-of-sample model evaluation.

#### Model building

Model development and evaluation followed the recommendations from the transparent reporting of a multivariable prediction model for individual prognosis or diagnosis (TRIPOD) statement.^46^ Model development was performed with a supervised machine-learning method, the least absolute shrinkage and selection operator (LASSO). With many predictors (*n* = 28), this approach is computationally more efficient than more ‘classical’ model selection methods such as subset selection, which use least squares to fit a linear model that contains a subset of the predictors.^47^ In contrast, LASSO is a penalised regression analysis method that can fit a model containing all predictors, and then perform regularisation and variable selection. Regularisation involves shrinking the sum of the absolute values of the regression coefficients; thus, LASSO (unlike other shrinkage methods such as ridge regression) can effectively exclude predictors from the final model by shrinking their coefficients to zero, i.e. performs variable selection.^48^ This regularisation approach helps mitigate overfitting and allows for a more parsimonious, interpretable and replicable model.^49^

#### Internal model validation

LASSO methods require the shrinkage hyper-parameter (λ) to be optimised for the data and model. In contrast to model parameter estimation methods in classical regression, LASSO algorithms do not yield standard errors and uncertainty intervals for estimated model parameters. For this reason, we apply the following procedure:

- Step 1, resample (with replacement) the model development data-set (*N* = 717) to generate a sample, *S*.
- Step 2, execute the LASSO procedure with ten-fold cross-validation on *S* to locate the optimal λ parameter that yields the minimum mean-squared error between predicted and actual AAO outcomes.
- Step 3, extract the model parameters given the optimal λ parameter.
- Step 4, repeat from step 1 1000 times.

We used the ‘cv.glmnet’ function from the R package ‘glmnet’ version 4.1-1^42^ for steps 2 and 3. After 1000 resample-fitting procedures, we collated all parameter estimates (coefficients) to examine which predictor variables were consistently retained, and estimated the variability in these coefficients (Supplementary Appendix 1, Section 3.2). We report these non-exponentiated coefficients as histograms, showing their distributions over 1000 resamples of the training set (Supplementary Appendix 1, Section 3.1).

**Fig. 1:**
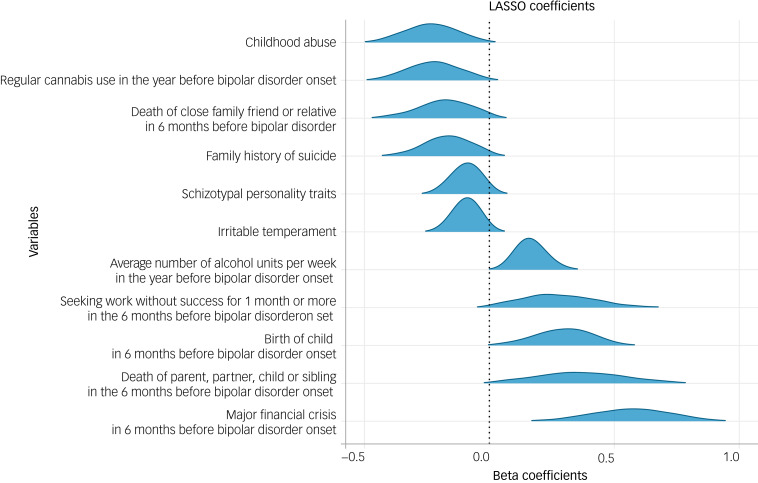
Density plots for the 11 predictors selected by cross-validated LASSO regression model on >90% of the 1000 resampling runs. Negative beta coefficients indicate an association with an earlier AAO, whereas positive coefficients represent an association with a later AAO. AAO, age at onset; LASSO, least absolute shrinkage and selection operator.

For inferential analysis, we ranked the number of times a predictor was included on each of the 1000 resampling runs/model fits. We then selected the predictors that were included on >90% of these runs. A 90% cut-off point was chosen pre-analysis as a limit that was sufficiently high enough to ensure predictors were reliably present in each model refit. Once we arrived at a selected set of predictors, to display effect sizes, we derived density plots for each predictor from the coefficients generated across the 1000 resamples (Fig. 1). We also report the most common (modal) coefficient value for each of the predictors present on >90% of reruns (Table 1).

**Table 1:** Non-exponentiated modal coefficients for each of the 11 predictors selected by the least absolute shrinkage and selection operator regression model on >90% of resampling runs

Predictors	Modal coefficients
Childhood abuse	−0.2855
Regular cannabis use in the year before bipolar disorder onset	−0.2765
Death of close family friend or relative in 6 months before bipolar disorder onset	−0.2435
Family history of suicide	−0.1385
Schizotypal personality traits	−0.1055
Irritable temperament	−0.0685
Average number of alcohol units per week in the year before bipolar disorder onset	0.1385
Birth of child in 6 months before bipolar disorder onset	0.2755
Death of parent, partner, child or sibling in the 6 months before bipolar disorder onset	0.3125
Seeking work without success for 1 month or more in the 6 months before bipolar disorder onset	0.3505
Major financial crisis in 6 months before bipolar disorder onset	0.4575

#### Model evaluation

In an exploratory internal validation, we applied the selected model to the held-out validation set (*n* = 305). The model generated predictions for bipolar disorder AAO for each case in the validation set. Model predictive performance was assessed with a calibration curve. Calibration refers to the agreement between observed AAO values in the validation set and predictions from the model, and can be represented graphically with predictions on the *x*-axis, observed outcome on the *y*-axis and a 45° line representing perfect calibration.^50^ We used a non-parametric locally weighted scatterplot smoothing algorithm (LOESS) to produce our calibration plot. LOESS is a form of regression that uses a weighted, sliding window (passing along the *x*-axis) average to calculate a line of best fit. The span parameter, which is the size of the sliding window, determines the amount of smoothing and was set to 0.3.^51^ Plotting the smoothed regression line allows us to examine calibration across the full range of predicted values.

## Results

### Sociodemographic and clinical characteristics of the sample

There were a total of 1022 participants. The sample is described with mean, s.d. and range for continuous variables (Table 2), and absolute and relative frequencies for categorical variables (Table 3).

**Table 2:** Means, s.d. and ranges for continuous measures in the total sample (*n* = 1022)

Variable	Mean (s.d., range)
Age at bipolar disorder onset	23.0 (9.86, 5–68)
Age at interview	45.5 (12.1, 18–83)
Alcohol units consumed per week in the year before bipolar disorder onset	14.5 (30.4, 0–350)
Trait neuroticism	15.7 (5.40, 0–23)
Schizotypal personality traits	20.8 (12.1, 1–58)
Cyclothymic temperament	7.08 (3.88, 0–12)
Depressive temperament	2.55 (2.31, 0–8)
Irritable temperament	2.62 (2.24, 0–8)
Hyperthymic temperament	3.63 (2.35, 0–8)
Anxious temperament	1.30 (1.14, 0–3)

**Table 3:** Absolute (*n*) and relative (%) frequencies for categorical variables in the total sample (*n* = 1022)

Variable		*n*	%
Diagnosis	Bipolar disorder type 1	630	61.6
	Bipolar disorder type 2	346	33.9
	Bipolar disorder schizoaffective	26	2.5
	Bipolar disorder not otherwise specified	20	2.0
Family history of affective disorders	No	177	17.3
	Yes	845	82.7
Family history of psychiatric disorders (other than affective disorders)	No	640	62.6
	Yes	382	37.4
Family history of suicide	No	837	81.9
	Yes	185	18.1
Education	Higher education	493	48.2
	No higher education	529	51.8
Highest occupation	Professional	556	54.0
	Non-professional	449	43.5
	Never worked	7	0.7
	Student	18	1.8
Childhood physical, sexual or emotional abuse	No	802	78.5
	Unknown	25	2.4
	Yes	195	19.1
Regular use of cannabinoids in the year before onset	No	914	89.4
	Yes	108	10.6
Regular use of non-prescription drugs (other than cannabinoids) in the year before onset	No	979	95.8
	Yes	43	4.2
Poor premorbid work adjustment	No	1018	99.6
	Yes	4	0.4
Poor premorbid social adjustment	No	1009	98.7
	Yes	13	1.3
Life events 6 months before bipolar disorder onset			
		
Serious illness, injury or assault	No	871	85.2
	Yes	151	14.8
Close relative suffered serious illness, injury or assault	No	893	87.4
	Yes	129	12.6
Death of parent, partner, child or sibling	No	956	93.5
	Yes	66	6.5
Death of close family friend or relative	No	902	88.3
	Yes	120	11.7
Separation from or break-up with partner	No	841	82.3
	Yes	181	17.7
Serious problem with a close friend, neighbour or relative	No	781	76.4
	Yes	241	23.6
Seeking work without success for 1 month or more	No	954	93.3
	Yes	68	6.7
Major financial crisis	No	926	90.6
	Yes	96	9.4
Problems with the police involving a court appearance	No	999	97.7
	Yes	23	2.3
Something of value was lost or stolen	No	970	94.9
	Yes	50	5.1
Birth of child	No	930	91.0
	Yes	92	9.0

### Predictors of bipolar disorder AAO

#### Model development

For >90% of the resampling runs, the cross-validated LASSO regression analysis (mean λ = 0.0182, s.d. = 0.00727) consistently selected 11 variables as predictors of AAO (Fig. 1). Of these 11 variables, the following six were associated with an earlier AAO: childhood abuse, regular cannabis use in the year before onset, death of a close family friend or relative in the 6 months before onset, family history of suicide, schizotypal personality traits and irritable temperament. Five variables were associated with a later AAO: the average number of alcohol units consumed per week in the year before onset; birth of a child in the 6 months before onset; death of parent, partner, child or sibling in the 6 months before onset; seeking work without success for 1 month or more in the 6 months before onset and a major financial crisis in the 6 months before onset. Of these 11 variables, some had partial correlation with one another as well as with non-chosen predictor variables, with effect sizes ranging small (±0.21) to moderate (0.68) (see Figs 2.2 and 2.3 in Supplementary Appendix 1, Section 2.4). The non-exponentiated modal coefficients for these 11 predictors are shown in Table 1. The full model with all predictors’ coefficients (not just those selected on >90% of resampling runs) can be found in Supplementary Appendix 1, Section 3.3.

#### Model internal validation on held-out samples

**Fig. 2:**
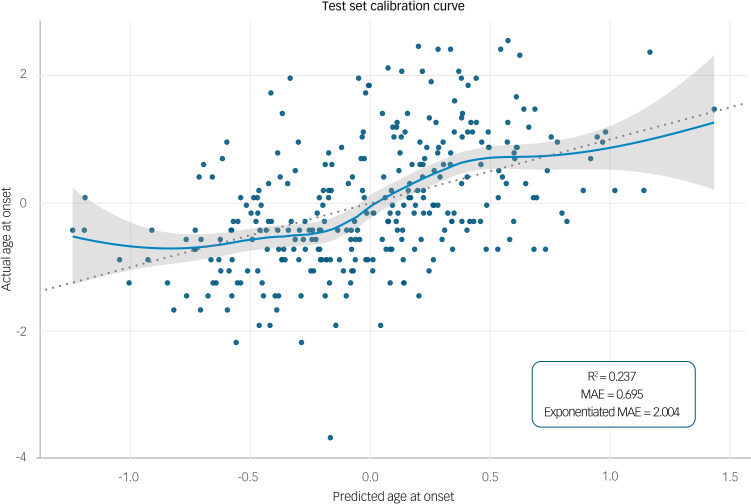
Calibration curve showing the agreement between observed outcomes and predictions, using the test set data. The dotted line represents ‘perfect model calibration’; the blue line is the calibration curve generated by our model with a locally weighted scatterplot smoother and 95% confidence intervals (grey); the blue scatter points are the observed data. Observed and predicted age at onset is shown on a natural logarithm scale. MAE, mean absolute error.

The model showed reasonable calibration when validated on the held-out test set with R^2^ = 0.237 and exponentiated mean absolute error (MAE) of 2.004. We chose exponentiated MAE as our metric for model accuracy as it is on the same scale as our outcome measure, AAO. Thus, the average absolute difference between the observed AAO and the predicted AAO values was approximately 2 years. This reasonable calibration can also be judged visually: as shown in Fig. 2, the predicted and observed AAO are similar, with approximately 90% of the model's confidence interval lying close to the 45° line.

#### *Post hoc* analysis

*Post hoc*, to ensure our model was reliable, we re-ran the full model building procedure by using elastic net penalised regression, which employs L1 and L2 regularisation, rather than LASSO, which uses L1 regularisation. The elastic net model selected the same 11 predictors as the LASSO regression, indicating that these features were stable across models, and showed comparable prediction accuracy (Supplementary Appendix 1, Section 3.4).

## Discussion

This is the first known study to comprehensively investigate a range of psychosocial predictors for bipolar disorder AAO. We found 11 variables that were reliably associated with bipolar disorder AAO. Six predicted an earlier AAO: childhood abuse, regular cannabis use in the year before onset, death of a close family friend or relative in the 6 months before onset, family history of suicide, schizotypal personality traits and irritable temperament. Five variables were associated with a later AAO: the average number of alcohol units consumed per week in the year before onset; birth of a child in the 6 months before onset; death of parent, partner, child or sibling in the 6 months before onset; seeking work without success for 1 month or more in the 6 months before onset and a major financial crisis in the 6 months before onset. We discuss these findings in the context of previous research, along with their implications for diagnosis, treatment and early intervention.

### Childhood abuse and individual-level characteristics

The variable that was associated with the earliest AAO was childhood abuse. This aligns with a large body of evidence indicating that maltreatment in childhood is associated with an earlier AAO,^8^ and is more common in individuals with bipolar disorder compared with healthy controls.^27^ As suggested by prior research, the trauma of childhood abuse may expedite the AAO of bipolar disorder.^27,52^ For instance, recent research (which has partial sample overlap with the current study) found that the path between childhood abuse and an earlier AAO was selectively explained by individuals’ mood instability.^53^ The authors suggest that mood instability – defined as rapid and intense fluctuations in affect – may bring forward illness onset in children who are vulnerable because of abuse, with increased mood instability developing into episodes of mania or depression.^54,55^ However, the causal nature of this relationship is yet unclear. It may be that behavioural difficulties and emotional dysfunction resulting from an early bipolar disorder AAO confer greater social and emotional vulnerability, which, in turn, has been identified as a major risk factor for childhood abuse.^56,57^ Thus, there may be a bi-directional relationship between mood instability and childhood abuse, with increased mood lability reducing an individual's resilience to childhood abuse, and/or *vice versa*, with childhood abuse increasing the likelihood for mood instability. Prospective longitudinal studies are needed to elucidate the precise nature of the relationship between mood instability, childhood abuse and how it relates to early-onset bipolar disorder.

The mediating effect of mood instability on the relationship between trauma and early-onset bipolar disorder parallels our finding that irritable temperament was associated with an earlier AAO. Irritable temperament has been positively associated with mood instability, borderline symptoms, impulsivity and grandiosity,^58^ as well as predicting manic symptoms.^59^ It has been suggested that irritable temperament forms part of a broader bipolar disorder spectrum and represents a prodromal phase of the disorder,^60^ and, as with mood instability, may accelerate the onset of manic or depressive episodes meeting diagnostic criteria. Indeed, the association between high levels of trait irritability and an earlier AAO may be an artifact of the increased likelihood of these individuals manifesting behavioural problems that are brought to the attention of psychiatric services, in turn making it more likely to receive an early (or timely) diagnosis. Indeed, the diagnosis of pre-pubertal bipolar disorder, which is prevalent in North America, requires irritability (not mania) as a core symptom for diagnosis.^61,62^

Irritability is not the only trait measure that appears to be predictive of an earlier AAO. Greater endorsement of schizotypal personality traits – including magical thinking, paranoid ideation, ideas of reference and social isolation or anxiety – was also associated with an earlier AAO in our model. As with irritability, it is thought that schizotypy represents a dimensional trait that indexes the genetic liability to bipolar disorder and forms part of the bipolar disorder spectrum.^63^ Although this is the first known study to specifically investigate schizotypal personality traits in relation to bipolar disorder AAO, previous research indicates that schizotypal traits are elevated in those with bipolar disorder and predict future hypomanic episodes.^64,65^ There is a growing body of evidence demonstrating that genes (e.g. variants of the catechol-o-methyltransferase gene) may interact with environmental factors, such as childhood abuse, to contribute to elevated levels of schizotypal traits in bipolar disorder.^66^ Additionally, greater genetic liability for schizophrenia in individuals with bipolar disorder has been associated with increased schizotypy scores.^67^ As our findings show a link between increased schizotypal traits and early AAO, this lends support to the idea that earlier- versus later-onset bipolar disorder may differ in genetic aetiology, and highlights the importance of recognising the role of genetic interactions with the psychosocial predictors in our model. Of note, however, schizotypal traits and irritable temperament were partially correlated with other personality traits not included in our final model (e.g. trait neuroticism, cyclothymic temperament, depressive temperament). These correlated, but not included, temperament traits may also be of predictive relevance. As dimensions of personality are known to be traits that cluster, this should be considered when interpreting the impact of personality traits on AAO.^59^

### Life experiences and familial risk

Pertinent to the discussion of gene-environment interactions, we found that ‘family history of suicide’ was predictive of an earlier AAO, which is in line prior research.^17,68^ A family history of suicide confers a stronger familial/genetic loading for suicidality and corresponding psychiatric disorders, which supports the view that genetics contribute to an increased vulnerability for an earlier AAO in bipolar disorder. This is consistent with evidence suggesting that early onset may be a more heritable form of bipolar disorder than late onset, with studies demonstrating differences in transmission patterns and more pronounced familial aggregation in early- compared with late-onset bipolar disorder.^10,52,69^ Beyond specific genetic influences, a family history of suicide can convey increased transgenerational risk based on intra-familial behavioural interactions and their associated stressors.^17,70^ This, in turn, influences family environment and reciprocal offspring resilience,^71^ hinting that epigenetic mechanisms may be at play in bipolar disorder AAO.^8,27,29^

Looking beyond childhood abuse and family suicide, our model also suggests that other negative early-life experiences may catalyse disorder onset. Namely, ‘the death of a close family friend or relative in the 6 months before bipolar disorder onset’ correlated with an earlier AAO. Within the psychological framework of the diathesis stress model, it is thought that early negative life events interact with predisposed vulnerability to precipitate disorder onset.^72,73^ Accordingly, evidence suggests a dose effect of exposure to stressful life events on the AAO of bipolar disorder, with a greater number of early stressors being significantly associated with an earlier AAO.^31,32^ In contrast to this, however, we found that that the following life events were associated with a later AAO: birth of a child, major financial crisis and/or death of parent, partner, child or sibling in the 6 months before bipolar disorder onset; as well as seeking work without success for 1 month pre-onset. Yet, the direction of these relationships is unclear. For instance, these are all life events that become more common with increasing age, which therefore confounds the direction of these associations with a later AAO. In support of this, we can see from Fig. 1 that the coefficient variability of these four variables is greater than the others (i.e. ‘flatter’ density plots), which suggests that the relationship between these life events and AAO may be less reliable than the other predictors in our model, and is likely weakened by age being a possible confound. Equally, there were small numbers of participants reporting these negative life events, which could have weakened the strength of their relationship to AAO. This slightly less robust association may also be partially attributed to the finding that the effect of life events on the emergence of bipolar disorder diminishes with age,^74^ perhaps because of the development of appropriate coping strategies or the presence of other neutralising life events.

### Substance use

The presence of stressful life events has also been associated with alcohol use,^75^ which was identified as a significant predictor in our model. Our model suggests that alcohol use correlates with a slightly later AAO. Previous research has found mixed results, with some studies demonstrating that premorbid alcohol use is significantly associated with an earlier AAO,^18,21,76^ whereas others have found an association with a later AAO.^20,77^ Similar to the age-dependent life events we discuss above, the relationship between alcohol use and an early bipolar disorder onset may be confounded by age restrictions on purchasing and accessing alcohol. Equally, however, it may be that increases in alcohol use masks the true AAO of bipolar disorder, with it being unclear if mood and behavioural disturbances are a consequence of incipient bipolar disorder or directly related to heavy alcohol use.^55^ Thus, individuals may not recognise their first incidence of impairment as specifically related to bipolar disorder, artificially inflating their reported AAO. Early prodromal symptoms, such as sleep disturbances and anxiety symptoms may be attributed to alcohol use rather than recognised as part of the clinical trajectory of early-stage bipolar disorder.^78^ Indeed, anxiety symptoms have been found to be both a cause and a consequence of heavy alcohol use, as well as a clinical precursor in bipolar disorder.^79–81^ This highlights that the relationship between alcohol consumption and bipolar disorder AAO is likely non-linear, and so our findings must be interpreted with caution.

Despite previously mixed findings regarding the relationship between AAO and alcohol use, our finding that alcohol use was associated with a later AAO and cannabis use was associated with an earlier AAO, directly corroborates previous research that controlled for age as a potential confound.^20^ Furthermore, evidence from systematic reviews and meta-analyses points toward a significant association between cannabis use and an earlier AAO in bipolar disorder,^82–84^ with results suggesting that cannabis use may trigger the onset of mania.^84–86^ The mechanism behind this effect is unclear, but it has been hypothesised that the principal ingredients in cannabis (tetrahydrocannabinol and cannabidiol) affect mood via their interaction with the endocannabinoid, dopamine and serotonin neurotransmitter systems.^87,88^ In contrast, alcohol use is thought to increase the risk for depressive rather than manic symptoms.^82,89^ This may help explain why increased alcohol use was not associated with an earlier AAO, as the presence of a manic episode is needed before a clinical diagnosis of bipolar disorder can be made.

### Strengths and limitations

This is the only known study that models a wide range of psychosocial markers of AAO in a large, well-characterised sample of participants with bipolar disorder. We use a novel machine-learning approach not previously employed when investigating bipolar disorder AAO, employing bootstrapping, k-fold cross-validation and a held-out validation set to ensure model robustness and reduce overfitting. Our model showed good calibration, indicating that we can be confident in its predictive validity.

There are, however, several methodological limitations that must be considered. Relating to model validation, we did not have an independent sample for external validation. Although we used a held-out test set, this was a subsample of our original data-set and therefore subject to the same limitations as the data used for model building. The most notable of these limitations is the cross-sectional retrospective nature of the study, and the cohort's limited generalisability. As the analyses are not based on prospective data, we cannot be sure of the direction of causality in our model, and it is unclear whether our predictors should be conceptualised as causal risks factors or as risk markers, i.e. a factor that is associated with an outcome but is not necessarily its cause.^90^ Although, as with many psychiatric illnesses, it is likely that the relationships are bidirectional and symbiotic. Retrospective studies are also subject to recall bias, which undermines the reliability of self-reported AAO. This was mitigated by referring to medical case notes rather than relying solely on self-report. Yet, it has been suggested that people with bipolar disorder may be more likely to recall depressive compared with manic episodes, or even fail to recognise hypomanic episodes pre-diagnosis as pathological.^91,92^ This introduces biases into individuals’ recall of their bipolar AAO. Additionally, as the sample was skewed toward a younger age at study entry (average age of 46 years), late-onset bipolar disorder may have been underreported, thus weakening the reliability of our model. Moreover, a bipolar disorder diagnosis in older age may be masked or missed in favour of more prevalent later-life disorders with psychiatric symptoms (e.g. frontotemporal dementia), further obscuring the true rate of late-onset bipolar disorder. Therefore, as a gold standard, future research investigating bipolar disorder AAO should aim to employ prospective longitudinal methodologies.

Furthermore, the included personality trait predictors had partial correlation with other personality variables not chosen in our final model. Thus, schizotypal personality traits and irritable temperament may not be the most valuable personality predictors *per se*, but rather represent the predictive importance of a clustering of other personality variables, such as high trait neuroticism and cyclothymic, depressive and anxious temperaments. Additionally, there are other theoretically driven potential psychosocial predictors that would have been interesting to include in our modelling. This includes information on pre-onset smoking and suicide attempts, as well as sleep and circadian rhythms, mood lability and premorbid anxiety, which are known to be important in the manifestation and prodromal stages of bipolar disorder.^93,94^ Additionally, given the likely role of gene×environment interactions, including genetic data in future analyses would help to elucidate aetiological mechanisms.

In conclusion, our study sheds light on the importance of several psychosocial markers for bipolar disorder AAO. Identifying these predictors provides is a further step toward understanding key processes in the aetiology of this heterogeneous psychiatric disorder. Our findings suggest that bipolar disorder AAO is likely catalysed via an interplay of genetic susceptibility, individual-level personality traits and exposure to negative life events and trauma in childhood. The identified predictor variables can be used to stratify individuals already at high-risk for bipolar disorder (e.g. offspring of parents with bipolar disorder) into likely AAO groups. Defining these AAO subgroups can help guide treatment provision and streamline approaches to early intervention. Future research should aim to externally validate our model in prospective, phenotypically detailed cohorts.

## Data Availability

The R code used for data pre-processing and analysis is openly available via the Open Science Framework. The data used in this study is not openly available as participants did not agree for their data to be shared publicly. For appropriately qualified researchers, however, data access may be granted upon reasonable request, and those interested should contact the BDRN.

## Supplementary material

For supplementary material accompanying this paper visit http://doi.org/10.1192/bjo.2022.536. click here to view supplementary material
