# Supplementary material for: Psychosocial markers of age at onset in bipolar disorder: a machine learning approach
Source: BJPsych Open. 2022 Jul 18;8(4):e133. doi: 10.1192/bjo.2022.536 (PMC9344222; doi:10.1192/bjo.2022.536)
Supplement: Supplementary file 1 [file S2056472422005361sup001.docx]

Supplement

Table of Contents

[1 Measures 2](#_Toc100748818)

[1.1 Candidate Predictors 2](#_Toc100748819)

[2 Data Analysis 5](#_Toc100748820)

[2.1 R packages 5](#_Toc100748821)

[2.2 Missingness: Comparing samples with vs. without missing variables removed 6](#_Toc100748822)

[2.3 Data Transformation 7](#_Toc100748823)

[2.4 Variable Correlation 9](#_Toc100748824)

[3 Results 11](#_Toc100748825)

[3.1 Histograms of non-exponentiated coefficients 11](#_Toc100748826)

[3.2 Predictors included on resampling runs 15](#_Toc100748827)

[3.3 Modal coefficient values for all predictors 17](#_Toc100748828)

[4 References 19](#_Toc100748829)

# Measures

## Candidate Predictors

All predictors were selected based on their practicality and potential relevance to BD AAO. Twenty-eight predictors were considered.

#### Psychiatric Family History

Participants were asked if they had a family history of (i) affective disorders, (ii) psychiatric disorders, and/or (iii) suicide. Answers were scored as ‘yes’, ‘no’ and ‘unknown’ for each of these three variables.

#### Childhood Abuse

The Childhood Life Events Questionnaire (CLEQ; (Upthegrove et al., 2015) was used to determine the presence of any known sexual and/or physical and/or emotional childhood abuse occurring before the age of 16 years. Answers were scored as ‘no known childhood physical, sexual or emotional abuse’ or ‘yes: experienced sexual, physical and or/emotional abuse before the age of 16’.

#### Alcohol units per week in year before onset

Participants were asked to enter the average number of units of alcohol they drank per week in the year before BD onset. If unknown, or if the participant did not use alcohol regularly, this was recorded as ‘NA’.

#### Drug Use

Participants were asked if they had ever regularly used cannabinoids or unspecified non-prescription drugs. ‘Regularly’ was defined as ‘persistently for one month, or at least once a week for >6 months of the year’. If participants answered ‘yes’ then they were asked if they cannabinoids or unspecified non-prescription drugs in the year before BD onset: yes, no and unknown.

#### Education

Highest level of educational attainment was recorded. Responses were grouped into ‘yes’ have attained higher education (degree or post-graduate degree), ‘no’ have no higher-education and ‘unknown’.

#### Work and Social Adjustment

The Modified OPCRIT Symptom Checklist Details and History questionnaire (Azevedo et al., 1999; Brittain et al., 2013) was used to assess work and social adjustment prior to illness onset:

1. **Poor premorbid work adjustment** refers to work history before onset of illness. Scored as ‘yes’ if the participant was unable to keep a job for >6 months; had a history of frequent job changes; wasn’t able to sustain a job expected by their educational level or training; persistently had a very poor standard of housework (housewives); failed to keep up with studies (students). Otherwise scored as ‘no’, ‘not applicable’ or ‘unknown’.
2. **Poor premorbid social adjustment** refers to social adjustment before onset of illness. Scored as ‘yes’ if the participant found difficulty entering or maintaining normal social relationships; showed persistent social isolation, withdrawal or maintained solitary interests prior to onset of symptoms; or participant having had no friends at school or only one casual friend. Participants who had several casual friends or good friends were scored as ‘no’. Otherwise, scores were ‘not applicable’ or ‘unknown’.

#### Trait Measures

Trait neuroticism, schizotypal personality traits, and five aspects of temperament were assessed as follows:

1. **Trait neuroticism.** Assessed using the Neuroticism subscale of the Eysenck Personality Questionnaire Revised (EPQ-R; (Eysenck & Eysenck, 1991). Scores range from 0 to 23, with higher total scores denoting higher levels of trait neuroticism.
2. **Schizotypal personality traits.** Assessed using the self-report Kings Schizotypy Questionnaire (KSQ; (Jones et al., 2000). This is a 63-item forced-choice (yes/no) questionnaire with seven subscales assessing schizotypy: recurrent illusions (2x subscales), social isolation, social anxiety, magical thinking, paranoid ideation, and ideas of reference. Higher total scores indicate higher levels of schizotypal personality traits.
3. **Temperament**. Evaluated using the Temperament Evaluation of Memphis, Pisa, Paris and San Diego Auto-questionnaire (TEMPS-A). This self-report questionnaire uses five subscales to assess cyclothymic, depressive, irritable, hyperthymic, and anxious temperament (Akiskal et al., 2005). Higher scores on each subscale indicate a greater number of features of the corresponding temperament.

*Life Events*

Eleven questions from the Brief Life Events questionnaire (BLEQ) were included as potential predictors (T. Brugha et al., 1985; T. S. Brugha & Cragg, 1990). These self-report questions referred to the six months before BD onset and were scored as ‘yes’ or ‘no’:

1. Did you suffer from a serious illness, injury, or assault?
2. Did a close relative suffer a serious illness, injury, or assault?
3. Did a parent, spouse/partner, child, or sibling of yours die?
4. Did a close family friend or relative die?
5. Did you have a separation due to marital difficulties or break off a steady relationship?
6. Did you have a serious problem with a close friend, neighbour or relative?
7. Were you seeking work without success for more than one month?
8. Did you have a major financial crisis such as losing the equivalent of three months’ income?
9. Did you have problems with the police involving a court appearance?
10. Was something you valued lost or stolen?
11. Did you or your wife/partner give birth to a child?

Three items from the BLEQ were excluded in our analysis. Item number seven – ‘In the 6 months prior to your illness onset, were you made redundant or sacked from your job?’ - was not included as we wanted to identify predictors of AAO, and not all participants were of legal working age at the time of their reported age at BD onset. Item 13 – ‘Do you think that anything happened which contributed to you becoming unwell? If yes, what was it?’ - and item 14 – ‘Do you think that there is anything that happened to you during your life which contributed to you becoming unwell? If yes, what was it?’ - were not included as these were free text answers and coding these into categories for quantitive analysis was beyond the scope of the current study.

# Data Analysis

## R packages

| **Package Name** | **Author(s)** | **Version** | **URL** |
| --- | --- | --- | --- |
| BSDA | Arnholt & Evans (2017) | 1.2.0 | <https://CRAN.R-project.org/package=BSDA> |
| car | Fox & Weisberg (2019) | 3.0-11 | <https://cran.r-project.org/web/packages/car/index.html> |
| caret | Kuhn (2019) | 6.0-84 | <https://CRAN.R-project.org/package=caret> |
| dplyr | Wickham et al. (2020) | 1.0.2 | <https://CRAN.R-project.org/package=dplyr> |
| feather | Wickham (2019) | 0.3.5 | <https://CRAN.R-project.org/package=feather> |
| forcats | Wickham (2020) | 0.5.0 | <https://CRAN.R-project.org/package=forcats> |
| ggforce | Pedersen (2021) | 0.3.3 | <https://CRAN.R-project.org/package=ggforce> |
| ggplot2 | Wickham (2016) | 3.3.2 | <https://ggplot2.tidyverse.org> |
| glmnet | Friedman et al. (2010) | 4.1-1 | ﻿<https://glmnet.stanford.edu> |
| ggridges | Claus O. Wilke (2021) | 0.5.3 | <https://CRAN.R-project.org/package=ggridges> |
| HDCI | Liu et al. (2017) | 1.0-2 | <https://cran.r-project.org/package=HDCI> |
| Hmisc | Harrell (2020) | 4.4-1 | <https://CRAN.R-project.org/package=Hmisc> |
| magrittr | Bache & Wickham (2020) | 2.0.1 | <https://CRAN.R-project.org/package=magrittr> |
| MASS | Venables & Ripley (2002) | 7.3-54 | [https://CRAN.R-project.org/package=MASS](https://cran.r-project.org/package=MASS) |
| moments | Komsta & Novomestky (2015) | 0.14 | <https://CRAN.R-project.org/package=moment> |
| naniar | Tierney et al. (2020) | 0.6.1 | <https://CRAN.R-project.org/package=naniar> |
| plyr | Wickham (2011) | 1.8.6 | <https://CRAN.R-project.org/package=plyr> |
| questionr | Barnier et al. (2020) | 0.7.4 | <https://CRAN.R-project.org/package=questionr> |
| rcompanion | Mangiafico (2021) | 2.4.1 | <https://CRAN.R-project.org/package=rcompanion> |
| readr | Wickham & Hester (2020) | 1.4.0 | <https://CRAN.R-project.org/package=readr> |
| recipes | Kuhn & Wickham (2020) | 0.1.15 | <https://CRAN.R-project.org/package=recipes> |
| tidyverse | Wickham et al. (2019) | 1.3.0 | ﻿<http://tidyverse.tidyverse.org> |

## Missingness: Comparing samples with vs. without missing variables removed

We compared the demographic and clinical characteristics of the dataset with and without missing values removed using X^2^ tests; Fisher exact test; and unpaired, 2-tailed t tests.

| **Variables** | | **Main Sample**  **(Missing Removed)**  **N = 1084** | | **Full Sample**  **N = 1468** | | **X^2^** | | |
| --- | --- | --- | --- | --- | --- | --- | --- | --- |
|  |  | **n** | **% of total sample** | **n** | **% of total sample** | **Test Statistic** | **df** | **p-value** |
| Diagnosis | BPI | 630 | 61.6 | ﻿889 | ﻿60.6 | 2.362 | 9 | 0.9844 |
|  | BPII | 346 | 33.9 | ﻿494 | ﻿33.7 |  |  |  |
|  | BP Schizoaffective | 26 | 2.5 | 45 | 3.1 |  |  |  |
|  | BP NOS | 20 | 2.0 | 40 | 2.7 |  |  |  |
| Family History of Affective Disorders | No | 177 | 17.3 | ﻿238 | 16.2 | 0.576 | 3 | 0.902 |
|  | Yes | 845 | 82.7 | ﻿1230 | 83.8 |  |  |  |
| Family History of Psychiatric Disorders | No | 640 | 62.6 | ﻿898 | 61.2 | 0.583 | 3 | 0.900 |
|  | Yes | 382 | 37.4 | ﻿570 | 38.8 |  |  |  |
| Family History of Suicide | No | 837 | 81.9 | ﻿1189 | 81.0 | 0.352 | 3 | 0.950 |
|  | Yes | 185 | 18.1 | ﻿279 | 19.0 |  |  |  |
| Education | Higher education | 493 | 48.2 | ﻿793 | 54.0 | 1.339 | 3 | 0.720 |
|  | No higher education | 529 | 51.8 | ﻿675 | 46.0 |  |  |  |
| Occupation | Professional | 552 | 54.0 | ﻿761 | 51.8 | 1.529 | 9 | 0.997 |
|  | Non-professional | 445 | 43.5 | ﻿669 | ﻿45.6 |  |  |  |
|  | Never worked | 7 | 0.7 | 13 | 0.9 |  |  |  |
|  | Student | 18 | 1.8 | 25 | 1.7 |  |  |  |
|  |  | **Main Sample**  **(Missing Removed)**  **N = 1084** | | **Full Sample**  **N = 1468** | | **X^2^** | | |
|  |  | **n** | **% of total sample** | **n** | **% of total sample** | **Test Statistic** | **df** | **p-value** |
| Regular use of cannabinoids in the year before onset | No | 914 | 89.4 | ﻿1308 | 89.1 | 0.075 | 3 | 0.995 |
|  | Yes | 108 | 10.6 | 160 | 10.9 |  |  |  |
| Regular use of non-prescription drugs in the year before onset | No | 979 | 95.8 | ﻿1394 | 95.0 | 1.016 | 3 | 0.798 |
|  | Yes | 43 | 4.2 | 74 | 5.0 |  |  |  |
| Poor premorbid work adjustment | No | 1018 | 99.6 | ﻿ ﻿1462 | 99.6 | 0.005 | 3 | 0.999 |
|  | Yes | 4 | 0.4 | 6 | 0.4 |  |  |  |
| Poor premorbid social adjustment | No | 1009 | 98.7 | 1442 | 98.2 | 1.059 | 3 | 0.787 |
|  | Yes | 13 | 1.3 | 26 | 1.8 |  |  |  |
| **Variables** |  |  |  |  |  | **Welch Modified Two-Sample t-Test** | | |
|  |  | Mean (SD) | Range | Mean (SD) | Range | **Test Statistic** | **df** | **p-value** |
| Age at Onset |  | 23.0 (9.86) | 5-68 |  |  | 0.157 | 27.383 | 0.876 |
| Age at Interview |  | 45.5 (12.1) | 18-83 |  |  | 0.084 | 26.39 | 0.934 |
| Alcohol Consumption |  | 14.5 (30.4) | 0-350 |  |  | -0.154 | 24.126 | 0.879 |

## Data Transformation

Our outcome variable Age at Onset (AAO) must be a positive real value, therefore we log-transformed the data such that log(AAO) has a mean of 3.0 and symmetric variance. Additionally, AAO was positively skewed (skewness = 1.34) and significantly non-normal (Shapiro-Wilk normality test W = 0.89172, p-value < 2.2e-16). Log transformation helped correct for non-normality as shown in Supplementary Figure 1 (reduced skewness: 0.29).

**Pre-transformation**

**Post-log-transformation**

Figure 2.1

## Variable Correlation

To address variable correlation, we have computed two correlation matrices: one including all 28 predictor variables, and one for the 11 ‘most important’ variables included in our final model.

Figure 2.2 Correlation matrix for the eleven variables included in our final model.

Figure 2.3 Correlation matrix for all variables.

# Results

## Histograms of non-exponentiated coefficients

We collated all parameter estimates (coefficients) to examine which predictor variables are consistently retained and estimated the variability in these coefficients. We report these non-exponentiated coefficients as histograms, showing their distributions over 1000 resamples of the training set.

## Predictors included on resampling runs

| **Predictors** | **N Resampling Runs** |
| --- | --- |
| Avg. number of alcohol units per week in the year before BD onset | 1000 |
| Major financial crisis in 6m prior to BD onset | 999 |
| Birth of child in 6m prior to BD onset | 992 |
| Childhood abuse | 987 |
| Irritable Temperament | 965 |
| Regular Cannabis use in the year before BD onset | 960 |
| Death of parent, partner, child, or sibling in the 6m prior to BD onset | 959 |
| Schizotypal personality traits | 948 |
| Family history of suicide | 943 |
| Seeking work without success for one month or more in the 6m prior to BD onset | 937 |
| Death of close family friend or relative in 6m prior to BD onset | 908 |
| Problems with the police involving a court appearance in 6m prior to BD onset | 885 |
| High trait neuroticism | 861 |
| Poor premorbid social adjustment | 853 |
| Cyclothymic Temperament | 841 |
| Hyperthymic Temperament | 835 |
| Family history of psychiatric disorders | 726 |
| Serious problem with a close friend, neighbour or relative in 6m prior to BD onset | 717 |
| Separation from or break-up with partner in 6m prior to BD onset | 694 |
| Regular drug use in the year before BD onset | 666 |
| Serious illness, injury, or assault in 6m prior to BD onset | 633 |
| Family history of affective disorders | 609 |
| Higher education | 607 |
| Close relative suffered serious illness, injury, or assault in 6m prior to BD onset | 580 |
| Something of value was lost or stolen in 6m prior to BD onset | 570 |
| Anxious Temperament | 560 |
| Depressive Temperament | 501 |
| Childhood abuse unknown | 479 |
| Poor premorbid work adjustment | 409 |

## Modal coefficient values for all predictors

| **Predictors** | **Modal Coefficients** |
| --- | --- |
| (Intercept) | 0.0855 |
| Avg. number of alcohol units per week in the year before BD onset | 0.1385 |
| High trait neuroticism | - 0.0835 |
| Cyclothymic Temperament | - 0.0835 |
| Depressive Temperament | ﻿- 0.0285 |
| Irritable Temperament | - 0.0685 |
| Hyperthymic Temperament | -0.0455 |
| Anxious Temperament | 0.0435 |
| Schizotypal personality traits | - 0.1055 |
| Family history of affective disorders | -﻿ 0.0215 |
| Family history of psychiatric disorders | ﻿- 0.0225 |
| Family history of suicide | ﻿- 0.1385 |
| Higher education | 0.0115 |
| Regular Cannabis use in the year before BD onset | ﻿- 0.2765 |
| Regular drug use in the year before BD onset | ﻿- 0.0705 |
| Poor premorbid work adjustment | ﻿- 0.1695 |
| Poor premorbid social adjustment | ﻿- 0.7495 |
| Childhood abuse | - 0.2855 |
| Childhood abuse unknown | - 0.0745 |
| Serious illness, injury, or assault in 6m prior to BD onset | - 0.0085 |
| Close relative suffered serious illness, injury, or assault in 6m prior to BD onset | - 0.1155 |
| Death of parent, partner, child, or sibling in the 6m prior to BD onset | 0.3125 |
| Death of close family friend or relative in 6m prior to BD onset | ﻿- 0.2435 |
| Separation from or break-up with partner in 6m prior to BD onset | 0.0525 |
| Serious problem with a close friend, neighbour or relative in 6m prior to BD onset | - 0.0845 |
| Seeking work without success for one month or more in the 6m prior to BD onset | ﻿ 0.3505 |
| Major financial crisis in 6m prior to BD onset | 0.4575 |
| Problems with the police involving a court appearance in 6m prior to BD onset | ﻿- 0.2945 |
| Something of value was lost or stolen in 6m prior to BD onset | ﻿ 0.0065 |
| Birth of child in 6m prior to BD onset | 0.2755 |

## Alternative Models

We have also rerun analysis (using the cv. glmnet’ package) employing elastic net (α = 0.5) and ridge regression (α = 0). We found that the elastic net model selected the same eleven features as the LASSO regression. Ridge regression resulted in a less parsimonious model with all 29 predictors included. The table below compares these models:

| **Model** | **Root Mean Squared Error** | **R-squared** |
| --- | --- | --- |
| LASSO | 0.881 | 0.237 |
| Elastic Net | 0.874 | 0.258 |
| Ridge | 0.881 | 0.251 |

# **References**

Akiskal, H. S., Mendlowicz, M. v., Jean-Louis, G., Rapaport, M. H., Kelsoe, J. R., Gillin, J. C., & Smith, T. L. (2005). TEMPS-A: Validation of a short version of a self-rated instrument designed to measure variations in temperament. *Journal of Affective Disorders*, *85*(1–2), 45–52. https://doi.org/10.1016/j.jad.2003.10.012

Arnholt, A., & Evans, B. (2017). *BSDA: Basic Statistics and Data Analysis* (R package version 1.2.0). https://CRAN.R-project.org/package=BSDA.

Azevedo, M. H., Soares, M. J., Coelho, I., Dourado, A., Valente, J., Macedo, A., Pato, M., & Pato, C. (1999). Using consensus OPCRIT diagnoses: An efficient procedure for best- estimate lifetime diagnoses. *British Journal of Psychiatry*, *175*(AUG.), 154–157. https://doi.org/10.1192/bjp.175.2.154

Bache, S. M., & Wickham, H. (2020). *magrittr: A Forward-Pipe Operator for R* (2.0.1). https://CRAN.R-project.org/package=magrittr

Barnier, J., Briatte, F., & Larmarange, J. (2020). questionr: Functions to make surveys processing easier. *CRAN*.

Brittain, P. J., Stahl, D., Rucker, J., Kawadler, J., & Schumann, G. (2013). A review of the reliability and validity of OPCRIT in relation to its use for the routine clinical assessment of mental health patients. *International Journal of Methods in Psychiatric Research*, *22*(2), 110–137. https://doi.org/10.1002/mpr.1382

Brugha, T., Bebbington, P., & Hurry, J. (1985). The List of Threatening Experiences: A subset of 12 life event categories with considerable long-term contextual threat. *Psychological Medicine*, *15*(1), 189–194. https://doi.org/10.1017/S003329170002105X

Brugha, T. S., & Cragg, D. (1990). The List of Threatening Experiences: the reliability and validity of a brief life events questionnaire. *Acta Psychiatrica Scandinavica*, *82*(1), 77–81. https://doi.org/10.1111/j.1600-0447.1990.tb01360.x

Claus O. Wilke. (2021). *ggridges: Ridgeline Plots in “ggplot2”* (0.5.3). https://CRAN.R-project.org/package=ggridges

Eysenck, H. J., & Eysenck, S. B. G. (1991). *Manual of the Eysenck personality scales (EPS adult)*. Hodder and Stoughton. https://openlibrary.org/works/OL771815W/Manual_of_the_Eysenck_personality_scales_%28EPS_adult%29

Fox, J., & Weisberg, S. (2019). An {R} Companion to Applied Regression, Third Edition. *Thousand Oaks CA: Sage.*, *September 2012*.

Friedman, J., Hastie, T., & Tibshirani, R. (2010). Regularization Paths for Generalized Linear Models via Coordinate Descent. *Journal of Statistical Software*, *33*(1), 1. /pmc/articles/PMC2929880/

Harrell, F. E. (2020). Hmisc: Harrell miscellaneous. *R Package Version 4.4-0*.

Jones, L. A., Cardno, A. G., Murphy, K. C., Sanders, R. D., Gray, M. Y., McCarthy, G., McGuffin, P., Owen, M. J., & Williams, J. (2000). The Kings Schizotypy Questionnaire as a quantitative measure of schizophrenia liability. *Schizophrenia Research*, *45*(3), 213–221. https://doi.org/10.1016/S0920-9964(99)00183-8

Komsta, L., & Novomestky, F. (2015). *moments: Moments, cumulants, skewness, kurtosis and related tests* (R package version 0.14). https://CRAN.R-project.org/package=moments

Kuhn, M. (2019). Package “caret” for R: Classification and Regression Training. In *R package version 6.0-84*.

Kuhn, M., & Wickham, H. (2020). *Recipes: Preprocessing Tools to Create Design Matrices* (R package version 0.1.15). https://CRAN.R-project.org/package=recipes

Liu, H., Xu, X., & Li, J. J. (2017). *HDCI: High Dimensional Confidence Interval Based on Lasso and Bootstrap* (1.0-2).

Mangiafico, S. (2021). *Rcompanion: Functions to Support Extension Education Program Evaluation* (R package version 2.4.1). https://CRAN.R-project.org/package=rcompanion

Pedersen, T. L. (2021). ggforce: Accelerating “ggplot2.” In *The Comprehensive R Archive Network* (0.3.3). https://CRAN.R-project.org/package=ggforce

Tierney, N., Cook, D., McBain, M., Fay, C., O’Hara-Wild, M., Hester, J., & Smith, L. (2020). Naniar: Data Structures, Summaries, and Visualisations for Missing Data. In *R Package*.

Upthegrove, R., Chard, C., Jones, L., Gordon-Smith, K., Forty, L., Jones, I., & Craddock, N. (2015). Adverse childhood events and psychosis in bipolar affective disorder. *British Journal of Psychiatry*, *206*(3), 191–197. https://doi.org/10.1192/bjp.bp.114.152611

Venables, W. N., & Ripley, B. D. (2002). Modern Applied Statistics with S Fourth edition by. In *World* (Vol. 53, Issue March).

Wickham, H. (2011). The split-apply-combine strategy for data analysis. *Journal of Statistical Software*, *40*(1). https://doi.org/10.18637/jss.v040.i01

Wickham, H. (2016). *ggplot2: Elegant Graphics for Data Analysis* (2nd ed.). Springer International Publishing. https://doi.org/10.1007/978-3-319-24277-4

Wickham, H. (2019). *feather: R Bindings to the Feather “API”* (0.3.5). https://CRAN.R-project.org/package=feather

Wickham, H. (2020). *forcats: Tools for Working with Categorical Variables (Factors). R package version 0.5.0.* (0.5.0). https://CRAN.R-project.org/package=forcats

Wickham, H., Averick, M., Bryan, J., Chang, W., McGowan, L., François, R., Grolemund, G., Hayes, A., Henry, L., Hester, J., Kuhn, M., Pedersen, T., Miller, E., Bache, S., Müller, K., Ooms, J., Robinson, D., Seidel, D., Spinu, V., … Yutani, H. (2019). Welcome to the Tidyverse. *Journal of Open Source Software*, *4*(43). https://doi.org/10.21105/joss.01686

Wickham, H., François, R., Henry, L., & Müller, K. (2020). A grammar of data manipulation [R package dplyr version 1.0.0]. In *Media*. https://CRAN.R-project.org/package=dplyr

Wickham, H., & Hester, J. (2020). *Readr: Read Rectangular Text Data* (R package version 1.4.0). https://CRAN.R-project.org/package=readr
